# Novel combinations of variations in KCNQ1 were associated with patients with long QT syndrome or Jervell and Lange-Nielsen syndrome

**DOI:** 10.1186/s12872-023-03417-2

**Published:** 2023-08-12

**Authors:** Nongnong Zhao, Zhengyang Yu, Zhejun Cai, Wenai Chen, Xiaopeng He, Zhaoxia Huo, Xiaoping Lin

**Affiliations:** 1 Department of Cardiology, Second Affiliated Hospital, Zhejiang University School of Medicine, Hangzhou, 310009 Zhejiang China; 2 Yuyao People’s Hospital of Zhejiang Province, Yuyao, Ningbo, 315400 Zhejiang China; 3 Experimental Teaching Center, School of Basic Medical Sciences, Zhejiang University, 866 Yuhangtang Road, Hangzhou, 310058 Zhejiang China

**Keywords:** Jervell and Lange-Nielsen Syndrome, Long QT syndrome, Genetic variants, KCNQ1, Compound heterozygosity

## Abstract

**Objectives:**

Long QT syndrome (LQTS) is one of the primary causes of sudden cardiac death (SCD) in youth. Studies have identified mutations in ion channel genes as key players in the pathogenesis of LQTS. However, the specific etiology in individual families remains unknown.

**Methods:**

Three unrelated Chinese pedigrees diagnosed with LQTS or Jervell and Lange-Nielsen syndrome (JLNS) were recruited clinically. Whole exome sequencing (WES) was performed and further validated by multiplex ligation-dependent probe amplification (MLPA) and Sanger sequencing.

**Results:**

All of the probands in our study experienced syncope episodes and featured typically prolonged QTc-intervals. Two probands also presented with congenital hearing loss and iron-deficiency anemia and thus were diagnosed with JLNS. A total of five different variants in KCNQ1, encoding a subunit of the voltage-gated potassium channel, were identified in 3 probands. The heterozygous variants, KCNQ1 c.749T > C was responsible for LQTS in Case 1, transmitting in an autosomal dominant pattern. Two patterns of compound heterozygous variants were responsible for JLNS, including a large deletion causing loss of the exon 16 and missense variant c.1663 C > T in Case 2, and splicing variant c.605-2 A > G and frame-shift variant c.1265del in Case 3. To our knowledge, the compound heterozygous mutations containing a large deletion and missense variant were first reported in patients with JLNS.

**Conclusion:**

Our study expanded the LQTS genetic spectrum, thus favoring disease screening and diagnosis, personalized treatment, and genetic consultation.

## Introduction

Congenital long QT syndromes (LQTS) is one of the first discovered hereditary ion channel diseases characterized by prolonged QT intervals on surface electrocardiography (ECG) as well as clinical manifestations such as syncope and sudden cardiac death (SCD) due to torsade de pointes or polymorphic ventricular tachycardia. The genetic variants responsible for LQTS disrupt the normal structure and function of ion channel proteins which play critical roles in action potential activities in cardiomyocytes and other cell types. Currently, the estimated prevalence of LQTS is around 1/2000[1] and is considered the most common cause of sudden cardiac death in youth [2, 3]. According to the inherent pattern, there are two conditions of LQTS. The most common form, so-called Romano-Ward syndrome (RWS), is typically inherited as an autosomal-dominant trait; meanwhile the rare form of Jervell-Lange-Nielson syndrome (JLNS) is inherited recessively. To date, it is reported that the three definite genes (KCNQ1, KCNH2, SCN5A) are responsible for 75% ~ 80% of LQTS [4–6]. However, in the remaining 20–25%[7] of patients with a definite clinical diagnosis of LQTS, the pathological genetic variant remains to be identified [8]. Expect above mentioned three definitive genes, more than half of the suspected genes still have insufficient evidence to prove their causality [8]. This study reports 3 cases of LQTS or JLNS that have been well characterized clinically and genetically. Novel genetic variations have been identified in our study, causing the pathogenesis of LQTS. What’s more, it is the first to report the compound heterozygous combination of genetic alterations, including a large deletion and a missense variant to our knowledge. Our study will certainly expand the spectrum of genetic variations responsible for LQTS, thus favoring its screening and diagnosis, personalized treatment, and genetic consultant.

## **Objects and methods**

### Research objects and clinical evaluation

Written informed consent was obtained from all the subjects or their legal guardians. Complying with the Helsinki Declaration, study was approved by the Ethics Committee of the second affiliated hospital of Zhejiang university school of medicine. The clinical data, including a complete medical history, family history, lab tests, and image studies, were systematically reviewed. A standard 12-lead ECG examination was performed on the probands and their available family members. The QT interval was measured from lead II or lead V5 and corrected with heart rate through Bazett’s formula QTc = QT/RR^1/2^. The diagnosis was established if the patients met at least one of the following conditions [9]: Schwartz score ≥ 3.5[10] excluding secondary LQTS; at least one pathogenic genetic variant was identified;12-lead electrocardiogram showed QT_C_≥500ms (excluding secondary LQTS); when QT_C_ was 480-490ms, symptoms as unexplained syncope was also presented, a pathogenic variant may be present or not.

### Mutational analysis

#### Whole exome sequencing

Genomic DNA was extracted from peripheral blood lymphocytes by standard procedures using QIAamp DNA Blood Midi Kit (Qiagen, Hilden, Germany). DNA quality and concentration were examined by NANOdrop2000 (Thermo Scientific, United States) and were fragmented by Bioruptor Pico to generate a paired-end library (200–250 bp). The whole exome was captured using the Sureselect Human All Exon V7 kit (Agilent Technologies, USA). The final purified DNA libraries were sequenced using the Illumina NovaSeq 6000 platform (Illumina, USA) as PE 150 bp reads, providing an average coverage depth for each sample of at least 100-fold. Quality control was performed before proceeding to the next step.

#### Data filtering and bioinformatics analysis

Adapter and low-quality reads were removed from raw sequencing data using AfterQC [11]. High-quality reads were then aligned to the reference sequence (hg19) using Burrows-Wheeler Aligner (BWA) [12]. The final BAM files were used for variant calling. Single nucleotide variants (SNVs), insertions, and deletions were screened using GATK (https://software.broadinstitute.org/gatk) [13]. All SNVs and indels were filtered and estimated via multiple databases, including 1000 Genomes (1000 human genome dataset), Genome AD (Genome Aggregation Database dataset), and ExAC (The Exome Aggregation Consortium dataset). In silico tools were used to predict the effect of missense variants. The Human Gene Mutation Database (HGMD) and Clinvar Database were used to screen mutations reported in published studies. Pathogenic variants are assessed under the protocol issued by ACMG [14]. All potential pathogenic variants were validated using conventional Sanger sequencing. Multi-species alignments of interested regions were performed using Clustal Omega online software(http://www.ebi.ac.uk/Tools/msa/clustalo/). PSIPRED (http://bioinf.cs.ucl.ac.uk/psipred/) was used for the in-silico analysis of the secondary structures of proteins affected by interested variants.

#### MLPA

Multiplex Ligation dependent Probe Amplification (MLPA) was performed using the SALSA MLPA Probemix P114 Long-QT kit (MRC-Holland, Amsterdam, the Netherlands) and according to the MRC-Holland protocol [15].

## Result

### Case 1

**Fig. 1:**
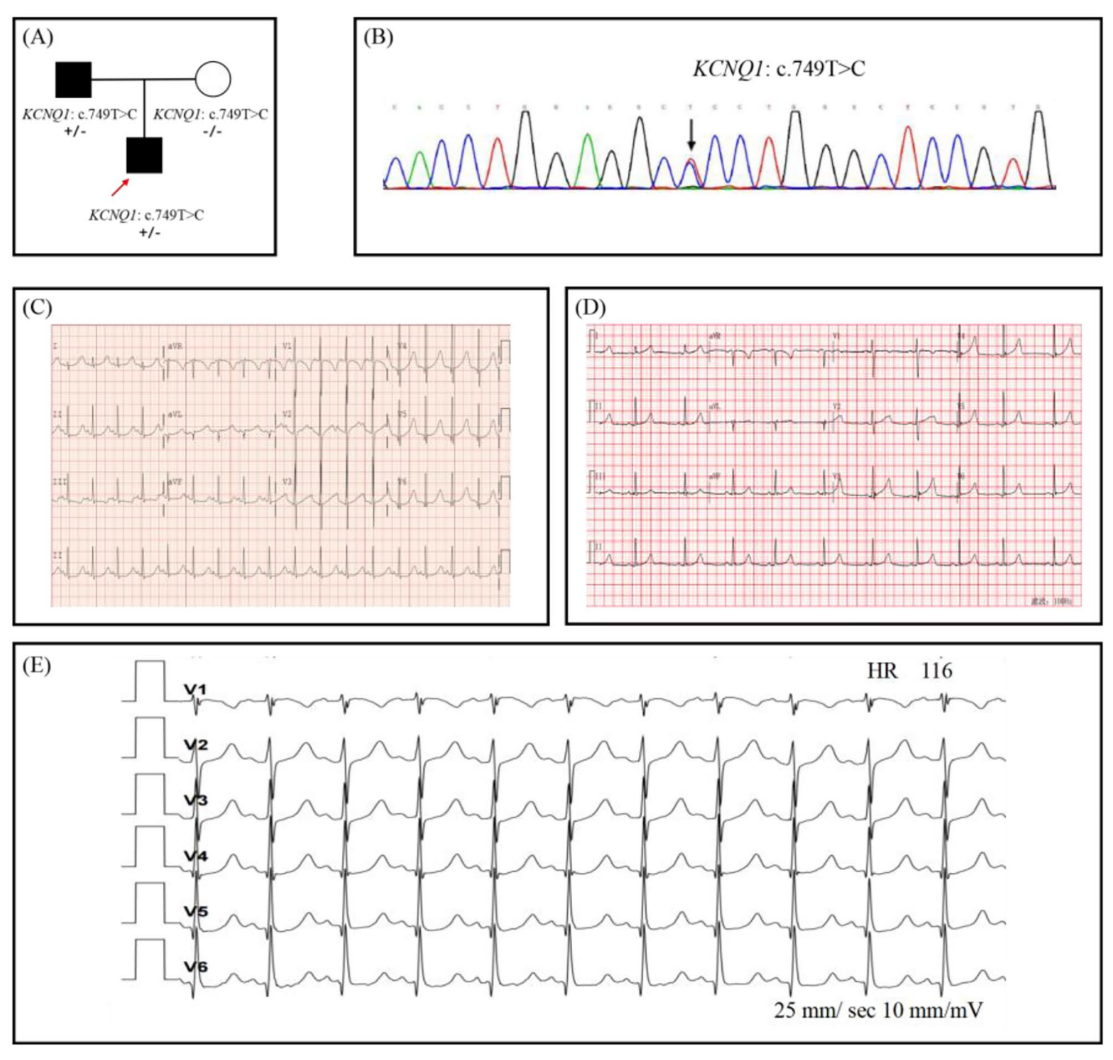
**(A)** Pedigree of Case 1. Arrow indicates the proband; squares indicate male family members; circles indicate female members; black filled indicate family members diagnosed with LQTS; **(B)** KCNQ1 c.749T > C were identified through WES and validated with Sanger sequencing; **(C)** The ECG of the proband in Case 1 before the β-blocker therapy showed the prolonged QTc interval(577ms); **(D)** The ECG of the proband showed significantly shortened QTc interval after propranolol therapy; **(E)** Treadmill exam of the proband’s father showed prolonged QTc up to 550ms at 3 min of the recovery phase

The proband, a 13-year-old boy (Fig. 1(A)), had experienced four episodes of syncope and seizure triggered by vigorous exercise or stress since the age of 6. He was first admitted to the neurology department for the evaluation of possible epilepsy. ECG found profound, prolonged QTc up to 577 ms (Fig. 1(C)). Echocardiography exam excluded any cardiac structure abnormalities. He was treated with maximal tolerated propranolol and remained asymptomatic. His QTc shortened to the normal range since propranolol treatment (Fig. 1(D)). His parents had normal QTc intervals upon regular ECG (father 444 ms and mother 425 ms, respectively). However, a treadmill exam of his father presented with prolonged QT_C_ up to nearly 550ms at 3 min of the recovery phase(Fig. 1(E)). Thus the diagnosis of LQTS was also suspected [16].

**Fig. 2:**
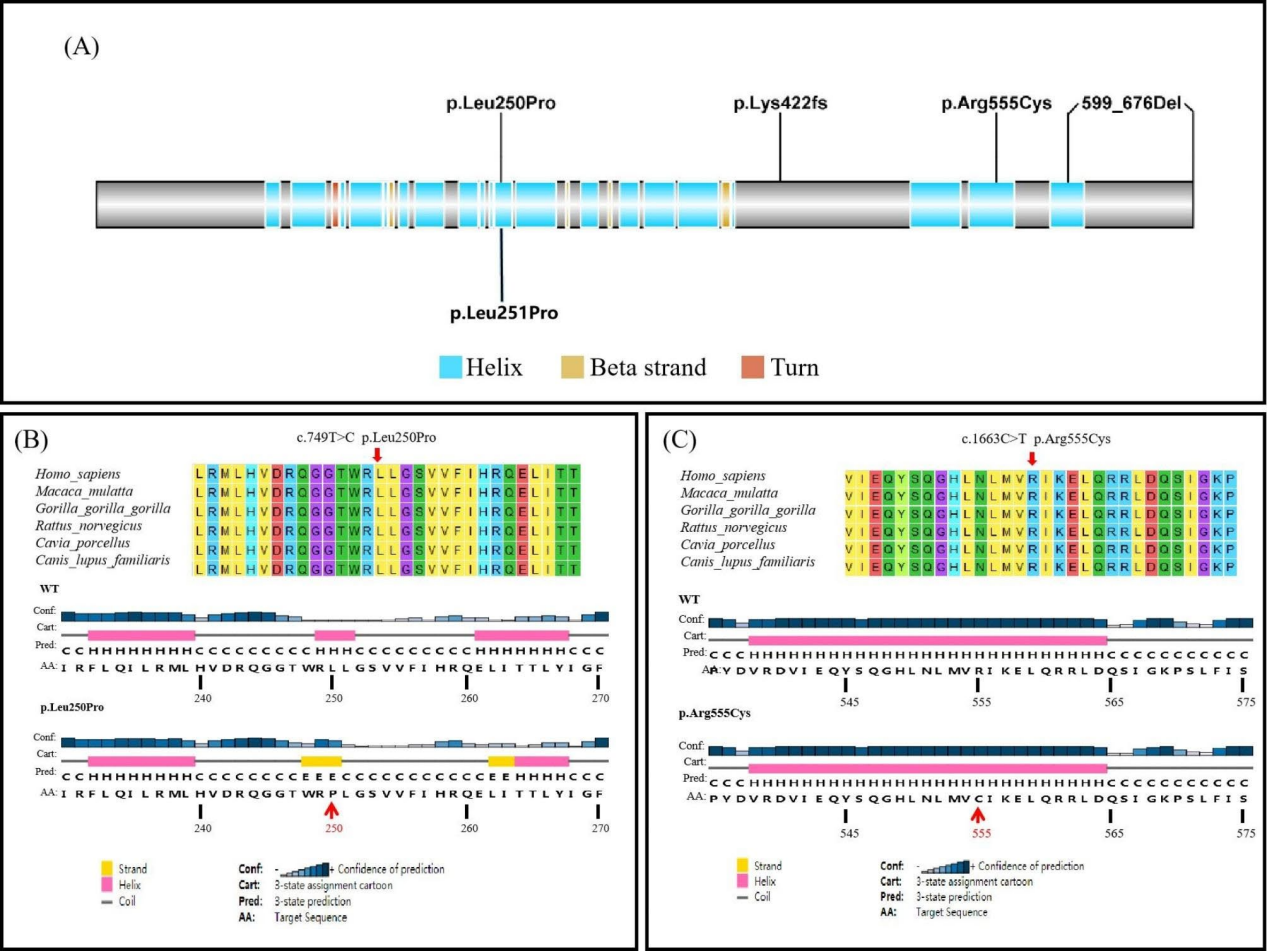
**(A)** The illustration indicated the amino acid changes in KCNQ1 found in our study. **(B)** KCNQ1 p.Leu250Pro was located in a highly conserved region across all available species evolutionally (upper). Altered secondary structure of p.Leu250Pro mutant protein was predicted when compared with the wild type (the altered residue was shown with red arrow) (lower). **(C)** KCNQ1 p.Arg555Cys was also located in a highly conserved region across all available species evolutionally (upper). No significant alteration of the secondary structure of p.Arg555Cys mutant protein was predicted when compared with the wild type (lower, the altered residue is shown with red arrow) (lower)

A heterozygous missense variant of c.749T > C(p.Leu250Pro)in KCNQ1 (Fig. 1(B)) was identified in the proband by initial WES and later confirmed to be transmitted from his father (Fig. 1 (A)). This variant has been reported in a patient with clinical features of LQTS [17], and it was extremely rare and absent in known human genetic variation databases (eg.1000 Genomes, GenomeAD, ExAC). In silico tools, including SIFT, Mcap, and Polyphen-2, predicted this variant of KCNQ1 to be a deleterious allele. KCNQ1 p.Leu250Pro was located in the S4–S5 linker, a functional domain of voltage-gated potassium channel mediating the interaction of S4 and S6(Fig. 2(A)), and was highly conserved evolutionally across all available species.(Fig. 2(B)). Computational simulation programs predicted altered secondary protein structure due to this rare variant (Fig. 2 (B)). This variant was extremely rare and absent in known human genetic variation databases (eg.1000 Genomes, GenomeAD, ExAC)(PM2), and it located in the S4–S5 linker, a well-established functional domain of voltage-gated potassium channel(PM1). In silico tools, including SIFT, Mcap, and Polyphen-2, predicted this variant of KCNQ1 to be a deleterious allele (PP3). The segregation phenomenon was observed in pedigree 1. The proband and his father, who carried the KCNQ1 c.749T > C mutation, showed a remarkable prolonged QT-intervals, while his mother had a normal QT-interval (PP1). Therefore, the novel KCNQ1 c.749T > C was segregated in the LQTS family and was regarded as likely pathogenic.

### Case 2

**Fig. 3:**
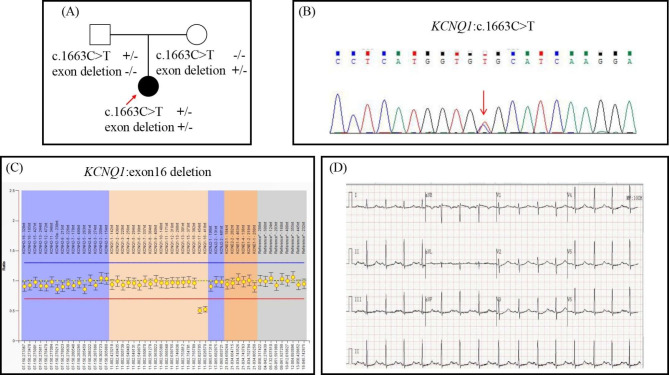
**(A)** Pedigree of Case 2. The proband carried compound heterozygous variants in KCNQ1, including c.1663 C > T and a large deletion causing loss of exon16. The missense variant was transmitted from her father, whereas the deletion was transmitted from her mother. **(B)** KCNQ1 c.1663 C > T were identified through WES and validated with Sanger sequencing; **(C)** MLPA probe confirmed a large deletion causing loss of KCNQ1 exon 16; **(D)** The ECG of the proband in Case 2 shows the significantly prolonged QTc interval(576ms)

The proband (Fig. 3(A)), a 20-year-old female, complained of recurrent exertional syncopal episodes since the age of 9. She suffered from sensorineural hearing loss since the age of 10 months and was assisted by ear aids since 14 months old. The 12-lead ECG showed a significantly prolonged QTc ranging from 545-576ms (Fig. 3(D)). In addition, her blood test indicated that she was suffering from iron-deficiency anemia (hemoglobin 92 g/L) without any obvious cause. Her echocardiography indicated no structure abnormalities. Thus, the diagnosis of JLNS was suspected according to the congenital hearing loss and significant QTc prolongation. She was treated with maximal tolerated propranolol and remained asymptomatic until now, and her QTc interval shortened to 496ms. Her parents were asymptomatic and had normal QTc intervals at rest (father 444ms and mother 426ms, respectively) and normal hearing.

A heterozygous missense variant was detected and a large deletion covering chr11:2868992–2,870,321 region in KCNQ1 was suspected in the proband by initial WES screening (Fig. 3 (B), (C)). The missense variant KCNQ1 c.1663 C > T was then validated by sanger sequencing and confirmed to be transmitted from the proband’s father (Fig. 3 (A)). The suspected large deletion was further validated by MLPA, leading to the complete loss of exon 16 in KCNQ1 (Fig. 3(C)). MLPA also detected the same large deletion in the proband’s mother. The KCNQ1 exon16 deletion, a null variation in KCNQ1 where LOF is a known mechanism of disease, led to exon skipping(PVS1) and it was at extremely low frequency human genetic variant databases(PM2). As a result, this exon skipping mutation was evaluated as likely pathogenic. KCNQ1 c.1663 C > T was located in exon 13, producing a substitution of arginine (Arg) with cysteine (Cys) at codon 555 (p.Arg555Cys) located in the C-terminus portion of the KCNQ1 protein. KCNQ1 p.Arg555Cys was located in a highly conserved region across multiple species (Fig. 2 (C)). The variant was extremely rare in human genetic variant databases (minor allele frequency(MAF) 0.000011 in genomAD; 0.000036 in ExAC, total population; <0.01 in 1000Genome) and was predicted to be likely pathogenic by Polyphen-2, SIFT, or Mcap. Although KCNQ1 c.1663 C > T (p.Arg555Cys) did not seem to alter the secondary structure in silico analysis (Fig. 2(C)), it was listed as a pathogenic variant concerning LQT phenotype in the ClinVar database. Hence, the compound heterozygous mutations, including a large deletion and a missense mutation, were presumed to lead to the pathogenesis of JLNS in the proband.

### Case 3

**Fig. 4:**
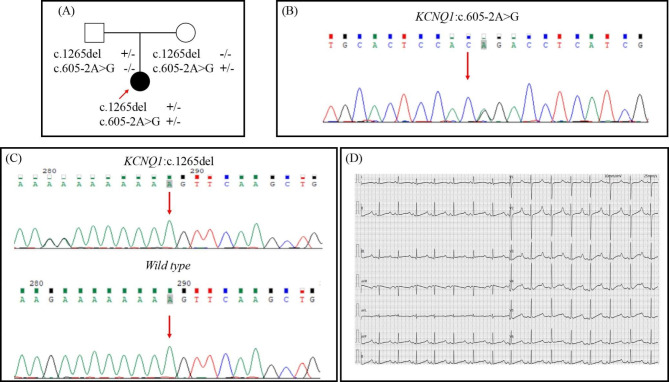
**(A)** Pedigree of the Case 3. The proband carries compound of heterozygous mutations of c.1265del and c.605-2 A > G. The missense mutation was transmitted from her mother and the frameshift variant was transmitted from her father. **(B)** DNA sequencing image for c.605-2 A > G in the KCNQ1; **(C)** DNA sequencing image for c.1265del in the KCNQ1; **(D)** The ECG of the proband in Case 3 shows the prolonged QTc interval(592ms)

The proband, a 5-year-old female (Fig. 4(A)), presented with syncope and seizure after vigorous activities and was born with severe sensorineural hearing loss, assisting with ear aid ever since. The 12-lead ECG showed a significantly prolonged QTc up to 591ms (Fig. 4(D)). She also suffered from iron-deficiency anemia and was supplemented with an oral iron solution and a normal cardiac structure showed by her echocardigraphy. Thus, the diagnosis of JLNS was made based on congenital hearing loss and significant QTc prolongation. She was treated with propranolol initiated at 0.5 mg/kg and kept on close follow-up. Her parents were asymptomatic and had normal QTc intervals at rest (father 425ms and mother 443ms, respectively) and normal hearing.

A heterozygous missense variant and a frame-shift variant in KCNQ1 were detected in the proband by initial WES screening (Fig. 4(B), (C)). The missense variant KCNQ1 c.605-2 A > G was then confirmed to be transmitted from the proband’s mother, whereases the frameshift variant c.1265del (p.Lys422fs) was transmitted from the proband’s father (Fig. 4(A)). The KCNQ1 c.605-2 A > G, a splicing variant, was extremely rare and characterized as likely pathogenic in the ClinVar database. Meanwhile, the frameshift variant c.1265del was also extremely rare and regarded as pathogenic in ClinVar. Nevertheless, it appeared to be the first time that both variants presented in the same patient, leading to the development of JLNS.

## Discussion

In the current study, three families with LQTS or JLNS were well characterized clinically, and novel variants or combinations in KCNQ1 were identified through WES screening. Well-established bioinformatics analysis and literature mining further indicated that these variations were pathogenic regrading LQTS or hearing loss phenotype in our cases.

The KCNQ1 encodes a subunit of the voltage-gated potassium channel, distributing in diverse types of cells, such as cardiomyocytes, inner ear cells, and neurons. Numerous variations in KCNQ1 have been identified to be associated with either LQTS (Romano-Ward syndrome) or JLNS [18]. JLNS was previously considered a result of homozygous mutations in KCNQ1. However, in recent years, evidence found that it could be caused by compound heterozygous mutations as well, largely enriching the genetic spectrum of the disease. Among them, only one study described a compound heterozygous mutations in KCNQ1 consisting of a large deletion spanning exons 7–10 and a frameshift mutation (c.1893dup; p.Arg632Glnfs*20) [19] To our knowledge, it was the first to report the compound heterozygous variations consisting of a large deletion and a missense variant in our case 2. In the previous study, KCNQ1_del_531-676 deletion [20] led to structural alteration of the potassium channel. A further electrophysiological study showed that, compared with the wild-type KCNQ1, the mutant with alternative truncated C-terminal yielded remarkably smaller activating currents and the absence of the tail currents. It may suggest that the exon 16 deletion of Case 2, leading to the deletion of amino acids 599 ~ 676, is pathological for this LQT1 case. For the KCNQ1 c.1663 C > T (p.Arg555Cys) variant, though no significant protein structure alteration has been detected in silicon, was demonstrated to result in reduced affinity of PIP2, which is necessary for regular potassium channel activity [21, 22]. Moreover, KCNQ1 protein was found to express in inner ear regions and the gastrointestinal tract, giving explanations for the defects of sensorineural hearing loss and iron deficiency anemia in JLNS cases.

KCNQ1 c.749T > A (p.Leu250His), the same location as in our case 1 (KCNQ1 c.749T > C, p.Leu250Pro), [23] displayed more prolonged QT intervals than other mutations in the vicinity S4-5 link and S5 area. Mutations of the S4-5 link and S5 area from LQTS patients exerted a loss of function effect, resulting in impaired activation rate and voltage-dependent activation and inactivation kinetics, as well as channel gating when co-assembled with auxiliary KCNE1 subunits. Hence, KCNQ1 c.749T > C might lead to prolonged QTc through a similar mechanism [22, 24]. In the state of stress or exercise, norepinephrine could regulate the IKs, a vital role in the ventricular repolarization phase. Norepinephrine binds to the β-receptor, a member of the G protein-coupled receptors family, and makes the concentration of cAMP. Subsequently, the protein kinase A(PKA) is activated and then binds with phosphodiesterase and phosphatase together to assemble a complex. This complex makes the PKA able to phosphorylate KCNQ1 and regulate IKs. One study reported that forskolin, the agonist of PKA, failed to activate the PKA in the individuals whose mutations located in the S4-S5 link, and subsequent phosphorylation of Kv7.1 was also significantly impaired. While the other mutations and wild type showed a dramatic activation [22]. That is, the mutations in C-loop could blunt PKA-mediated activation of channel activity, KCNQ1 c.749T > C, which is also located in the C-loop (S4-5 link), might lead to the maladaptation of cardiomyocyte to the rapid heart rate during repolarization phase. It might partly explain the reason why the father of the proband of Case 1 was likely to be a “concealed” LQTS patient, presenting normal QTc intervals in rest (444ms) but significantly prolonged QTc intervals provoked by exercise [25].It was reported that nearly half of LQT1 patients had a “conceal” QTc interval prolongation whereases the QTc intervals in LQT2 patients were relatively fixed, free from exercise and heart rate. A study showed that genotype-confirmed LQTS patients had a 10-fold higher cumulative probability (4%) of aborted cardiac arrest or SCD than genotype-negative family members (0.4%) [26]. The genotype-confirmed LQTS patients, though with normal QTc intervals in rest, also had a malignant prognosis compared to the general public and thus may benefit from early intervention. The mutations in nearby residues have also been reported to be associated with LQTS, including KCNQ1 c.733G > T, c.752T > C, and c.758 C > G. Co-expression of c.751T > C mutant, located near the mutation in Case 1, with the regulatory subunit of KCNE1 produced a remarkable reduction in the potassium currents amplitude, suggesting a dominant negative effect [27]. Hence, the KCNQ1 c.749T > C was considered as a pathological variant of our LQTS family.

The splicing variant of case 3 (KCNQ1 c.605-2 A > G (p.Asp20sp) was presumed to disrupt the normal splice and translate abnormal mRNA into pathological protein responsible for nonsense-mediated mRNA decay or pathological genotype. Previously, this alteration has been reported for several times [17, 28, 29], nevertheless, the functional study was lacking. Another variant, KCNQ1 c.1265del (p.Lys422fs), first reported in 1997 [30], has appeared in patients with LQTS or sinus bradycardia in different studies [31]. This frameshift variant was predicted to exert a loss of function effect due to protein truncation and nonsense-mediated mRNA decay [22]. Although these two variants have been separately reported, the heterozygous combination has not appeared in the literature.

Though potential pathogenic variants other than KCNQ1 was not found in our cases after careful screening, digenic mutations have been described in LQTS cases and were not rare. Recently, a rare form of KCNH2 and KCNE2 digenic mutations was reported in a LQTS patient [32]. However, unlike other multigenic mutation carriers, this patient did not present more severe clinical manifestation such as early onset of disease, much more prolonged QTc or more frequent ventricular tachyarrhythmias events [33], suggesting one mutation could be causal and the other one may be a modifier. Whereas other form of digenic mutations caused not only typical prolonged QTs related phenotype but also sinoatrial node dysfunction [34]. These studies indicated that recruiting of a more complete gene panel or whole exome sequencing was necessary as to fully reveal the predisposing genes and elucidate the mechanisms behind the phenotypic heterogeneity, thus aiding precised family screening, genetic consultation, risk stratification and clinical decision making [35].

β-blocker therapy played a critical role in the management of LQT1 patients due to the irresponsive of the mutant channel to sympathetic excitation [22]. A Meta-analysis reported that with the β-blocker treatment, the patients with QTc > 500ms still had a greater risk of cardiac events compared with the patients with QTc < 500ms [36]. LQTS patients may benefit from the shortened QTc intervals by avoiding cardiac events. That is the reason why after keeping away from exercise and being treated with propranolol, the proband of case 1 and case 2 have shorter QT intervals and manifestations in control. For the patients with ‘β-blocker failure’, left cardiac sympathetic denervation (LCSD) and ICD therapy should be taken into consideration, especially for the LQT1 patients. Besides, CRISPR/Cas9 is another potential therapeutic way, which can add, replace or delete the point mutation or replace the target mutant DNA sequence with the wild type and prepared DNA sequence. However, it is inefficient to edit the majority of cardiomyocytes [37] and possible to evoke an immune response harmful to edited cardiomyocytes and normal organizations [38]. In 2021, Steven M Dotzler et al.[39] reported the first proof-of-principle gene therapy, suppression-and-replacement (SupRep), for treating long QT syndrome at the genetic level. After the SupRep therapy, the IKs of iPSC-CM had no significant difference from the normal wild-type cardiomyocytes, suggesting that SupRep therapy is a potential therapeutic approach for LQTS.

## Conclusions

In summary, we identified novel combinations of pathogenic variants in KCNQ1 in three LQTS or JLNS families. Our study expanded the LQTS spectrum and thus facilitated future genetic consulting, disease screening, and personalized medicine. Patient-specific iPSC-CM-based research was warranted for further mechanistic study and therapeutic exploration.

## Data Availability

The raw sequence data reported in this paper have been deposited in the Genome Sequence Archive (Genomics, Proteomics & Bioinformatics 2021) in National Genomics Data Center (Nucleic Acids Res 2022), China National Center for Bioinformation / Beijing Institute of Genomics, Chinese Academy of Sciences (GSA-Human: HRA004325) that are publicly accessible at https://ngdc.cncb.ac.cn/gsa-human [40, 41].
